# In Vitro Performance of an Investigational Vibrating-Membrane Nebulizer with Surfactant under Simulated, Non-Invasive Neonatal Ventilation Conditions: Influence of Continuous Positive Airway Pressure Interface and Nebulizer Positioning on the Lung Dose

**DOI:** 10.3390/pharmaceutics12030257

**Published:** 2020-03-12

**Authors:** Federico Bianco, Elena Pasini, Marcello Nutini, Xabier Murgia, Carolin Stoeckl, Martin Schlun, Uwe Hetzer, Sauro Bonelli, Marta Lombardini, Ilaria Milesi, Marisa Pertile, Stefan Minocchieri, Fabrizio Salomone, Albert Bucholski

**Affiliations:** 1 Department of Preclinical Pharmacology, R&D, Chiesi Farmaceutici S.p.A., 43122 Parma, Italy; e.pasini@chiesi.com (E.P.); m.nutini@chiesi.com (M.N.); S.bonelli@chiesi.com (S.B.); m.lombardini@chiesi.com (M.L.); i.milesi@chiesi.com (I.M.); m.pertile@chiesi.com (M.P.); f.salomone@chiesi.com (F.S.); 2 Scientific Consultancy, 48640 Bilbao, Spain; xabi_murgia@hotmail.com; 3 PARI Pharma GmbH, 82319 Starnberg, Germany; Carolin.Stoeckl@pari.com (C.S.); Martin.Schlun@pari.com (M.S.); Uwe.Hetzer@pari.com (U.H.); Albert.Bucholski@pari.com (A.B.); 4 Division of Neonatology, Cantonal Hospital Winterthur, 8401 Winterthur, Switzerland; Stefan.Minocchieri@ksw.ch

**Keywords:** eFlow nebulizer, nebulized surfactant, aerosol, non-invasive ventilation, PrINT

## Abstract

Non-invasive delivery of nebulized surfactant has been a long-pursued goal in neonatology. Our aim was to evaluate the performance of an investigational vibrating-membrane nebulizer in a realistic non-invasive neonatal ventilation circuit with different configurations. Surfactant (aerosols were generated with a nebulizer in a set-up composed of a continuous positive airway pressure (CPAP) generator with a humidifier, a cast of the upper airway of a preterm infant (PrINT), and a breath simulator with a neonatal breathing pattern. The lung dose (LD), defined as the amount of surfactant collected in a filter placed at the distal end of the PrINT cast, was determined after placing the nebulizer at different locations of the circuit and using either infant nasal mask or nasal prongs as CPAP interfaces. The LD after delivering a range of nominal surfactant doses (100–600 mg/kg) was also investigated. Surfactant aerosol particle size distribution was determined by laser diffraction. Irrespective of the CPAP interface used, about 14% of the nominal dose (200 mg/kg) reached the LD filter. However, placing the nebulizer between the Y-piece and the CPAP interface significantly increased the LD compared with placing it 7 cm before the Y-piece, in the inspiratory limb. (14% ± 2.8 vs. 2.3% ± 0.8, nominal dose of 200 mg/kg). The customized eFlow Neos showed a constant aerosol generation rate and a mass median diameter of 2.7 μm after delivering high surfactant doses (600 mg/kg). The customized eFlow Neos nebulizer showed a constant performance even after nebulizing high doses of undiluted surfactant. Placing the nebulizer between the Y-piece and the CPAP interface achieves the highest LD under non-invasive ventilation conditions.

## 1. Introduction

The first attempts to deliver exogenous surfactant to preterm infants date back to 1964 and consisted of nebulizing synthetic DPPC microaerosols into the atmosphere of neonatal incubators [1]. These attempts were obviously unsuccessful and moved the field towards the development of intratracheal surfactant instillation protocols [2], which later gained clinical approval for the treatment of neonatal Respiratory Distress Syndrome (RDS). Surfactant replacement therapy remains a first-line treatment for RDS that improves survival and reduces the incidence of pneumothorax [3]. Nevertheless, it is considered an invasive therapy, often requiring positive pressure ventilation, which is considered as an important risk factor for developing Bronchopulmonary Dysplasia (BPD). To reduce the exposure of RDS babies to potentially harmful positive pressure ventilation, alternative instillation protocols have been developed such as Intubate-Surfactant-Extubate (InSurE) [4] and the Less Invasive Surfactant Administration (LISA) [5], which have become a common practice, particularly in Europe [3]. An alternative method that consist in the use of a Laryngeal Mask Airway (LMA) seems appealing but is still under investigation [6].

Surfactant replacement therapy by nebulization during non-invasive ventilation represents a truly non-invasive surfactant administration method, which, unfortunately, remains an unfulfilled goal of modern neonatal care. Several clinical studies have demonstrated the safety of nebulized surfactant in combination with continuous positive pressure ventilation (CPAP) [7,8,9,10,11]. Recently, Minocchieri et al. have demonstrated that *Poractant alfa* nebulized with a vibrating-membrane nebulizer reduces the need for intubation in 32^0^–33^6^ weeks gestational age (GA) infants with mild RDS, although no differences were observed in 29^0^–31^6^ weeks GA babies [11]. Although the efficacy of nebulized surfactant delivered during non-invasive ventilation cannot be concluded from these studies, these initial clinical experiences served to gain awareness of the complexity of delivering aerosols to preterm neonates and to further identify the factors affecting lung deposition in preterm infants [12].

Delivering nebulized medications to preterm neonates during non-invasive ventilation is one of the most challenging scenarios for aerosol medicine. Preterm infants are forced nasal breathers, have small airways, very low lung volumes and breathe at high respiratory rates [13]. These intrinsic and unchangeable variables promote a high impaction of surfactant in the upper airways, limit the amount of aerosol-containing air entering the lungs, and reduce the lung residence time of aerosol particles. Other factors that may affect lung deposition include the choice of the nebulizer and its placement in the circuit, the non-invasive ventilation (NIV) modality and its settings, the choice of NIV interface, and the physicochemical characteristics of the formulation [12,14]. These variables are extrinsic to preterm babies and therefore could theoretically be optimized to improve lung deposition. Additionally, in the particular case of surfactant nebulization, the viscosity of the phospholipid suspension significantly affects the aerosol production rate and the particle size distribution of nebulizers [15,16], which may impair the long-term performance of the device [10].

In vitro studies provide an excellent platform to investigate the influence of extrinsic factors on the overall lung deposition in preterm infants. We have recently shown that a customized version of the eFlow Neos vibrating-membrane nebulizer (PARI Pharma, Starnberg, Germany) can successfully nebulize undiluted *Poractant alfa,* achieving a mass median particle diameter of 3 µm [17]. Additionally, the same device-surfactant combination placed between the Y-piece and the nasal prongs achieved a mean lung surfactant deposition of 14% (nominal dose of 200 mg/kg) during simulated non-invasive neonatal ventilation conditions, in a set-up composed of a CPAP generator with a humidifier, a cast of the upper airways of a preterm infant, and a breath simulator with a neonatal breathing pattern [17]. Taking advantage of the established set-up, our aim here was to investigate: 1) if using a nasal mask as a CPAP interface rather than nasal prongs could improve lung deposition; 2) if a different positioning of the nebulizer in the circuit could affect lung deposition; and 3) whether increasing the nominal dose from 200 to 600 mg/kg would achieve an proportionally increased surfactant lung dose.

## 2. Materials and Methods

### 2.1. Nebulizer Type and Surfactant Preparation

Five independent eFlow Neos vibrating-membrane nebulizers, customized to neonatal standards, were used in the present study. The nebulizer was controlled by the eVent-Neos controller (PARI Pharma, Starnberg, Germany). This neonate-focused nebulizer has been miniaturized to fit appropriately into non-invasive neonatal ventilation circuits and can nebulize undiluted surfactant at relatively high rates (~0.2 mL/min or 16 mg of *Porctant alfa*/min) [17].

All experiments were performed nebulizing the commercially-available, animal-derived surfactant preparation *Poractant alfa* (Curosurf^®^, Chiesi Farmaceutici, Parma, Italy). This surfactant is a natural preparation, extracted from porcine lungs, containing almost exclusively polar lipids, in particular, Phosphatidylcholine (PC, about 70 % of the total phospholipid content), and about 1 % of specific low molecular weight hydrophobic proteins SP-B and SP-C, at a phospholipid concentration of 80 mg/mL. *Poractant alfa* is ordinarily administered as an intratracheal bolus at a dose of 200 mg/kg.

### 2.2. Surfactant Aerosol Deposition Experiments in a Simulated Neonatal CPAP Circuit

**Figure 1:**
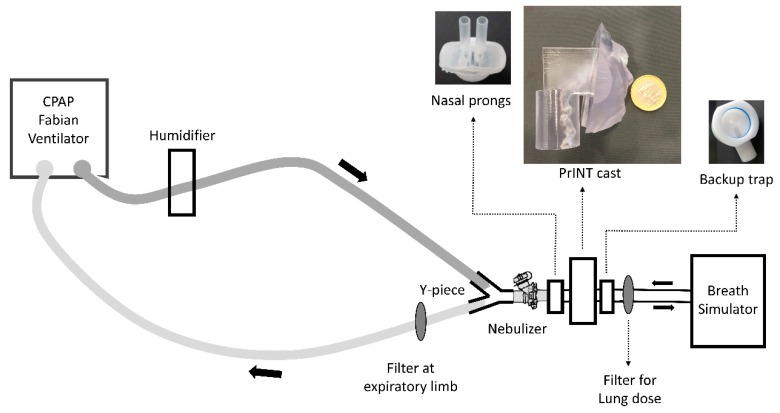
Scheme of the experimental set-up. CPAP, continuous positive airway pressure.

To investigate the deposition of nebulized surfactant during non-invasive neonatal ventilation, a clinical CPAP circuit was built (Figure 1). CPAP was generated using a neonatal ventilator (Fabian HFO, Acutronic, Zug, Switzerland) with a bias flow-rate of 5 L/min and a CPAP level of 5 cm H_2_O. The set-up included a cast of the upper airways of a premature infant (PrINT model; 32 weeks of gestation and 1750 g of body weight) [18] connected to a breath simulator (Compas 2, PARI Pharma, Starnberg, Germany) programmed with the breathing pattern of a premature infant with moderate RDS: rate of 70 bpm; tidal volume(V_T_) of 8.9 mL (5 mL/kg); inhalation/exhalation ratio of 40/60. The infant nose-throat cast was 3D printed (1zu1 prototypen, Dornibirn, Austria) as a solid substance (material: DSM water clear ultra 10122). Surfactant collection filters (PARI Filter PADs PZN: 00632160, Starnberg, Germany) were placed at the distal airway of the PrINT cast, to estimate the surfactant lung dose (LD), and at the expiratory limb of the CPAP system, to estimate the amount of surfactant aerosol exiting the CPAP circuit (exhalation filter). The temperature of the system was kept at 37 °C and the relative humidity was set at 90% ± 5 (MR 730, Fisher & Paykel Healthcare, Auckland, New Zealand). Before starting the experiments, the whole system was inspected for leak tightness.

To investigate how a different positioning of the nebulizer within the ventilation circuit could affect the LD and the overall aerosol deposition, two different CPAP circuits were assembled. In the first set of experiments, the Flexitrunk ventilation system (BC191, Fisher & Paykel Healthcare) was used with the nebulizer placed 7 cm away from the nasal mask (size M, Fisher & Paykel Healthcare) (Figure 1: position 1). In the second set of experiments, the Evaqua 2 infant respiratory care ventilation system (RT265, Fisher & Paykel Healthcare) was used. In this case, the nebulizer was placed between the Y-piece and the CPAP interface, close to the nose of the PrINT cast (Figure 1: position 2). Two different CPAP interfaces were tested: infant nasal mask (BC801-10, size M, Fisher & Paykel Healthcare) and nasal prongs (3520-10, Fisher & Paykel Healthcare). To achieve a tight seal between the interfaces and the PrINT cast, the nose area or the nostrils of the cast was silicon-coated. A backup trap was placed between the the PrINT cast and the inspiratory filter to collect the aerosol that impacts on the cast and moves forward as a liquid.

In each experiment, a volume of 4.37 mL of *Poractant alfa* at a concentration of 80 mg/mL was loaded into the nebulizer reservoir and continuously nebulized. This surfactant volume corresponds with a 200 mg/kg dose for a 1750 g infant. After nebulization of the full surfactant dose, the required nebulization time was annotated and all components of the system were carefully dissembled for phospholipid analysis. The LD was defined as the amount of surfactant collected within the filter placed at the distal airway of the PrINT cast. Besides, the amounts of surfactant deposited in the CPAP circuit, exhalation filter, backup trap, nasal prongs, as well as the surfactant remaining in the nebulizer, were also determined. The experiments representing each condition were repeated five times and the results are presented as the mean and standard deviation (SD) of the percentage of the deposited amount, considering a nominal dose of 200 mg/kg.

### 2.3. Dose-Dependent Deposition in a Simulated Neonatal CPAP Circuit

The same set-up described above but with the nebulizer placed between the Y-piece and the nasal prongs was used to conduct a dose-dependent deposition study. Three different *poractant alfa* doses were delivered: 2.18, 4.37 and 13.11 mL, which respectively correspond to 100, 200 and 600 mg/kg doses for a 1750 g infant. The experiments with 100 and 200 mg/kg doses were repeated 5 times, whereas those involving the 600 mg/kg dose were repeated 4 times. After the nebulization of the full surfactant dose, the required nebulization time was annotated. In the course of the nebulization experiments with the 600 mg/kg dose, the surfactant collecting filters were replaced each time after delivering 200 mg/kg. The results are presented as the mean and SD of the percentage of the deposited amount and as the mean of the total deposited phospholipid dose (in mg/kg).

### 2.4. HPLC Analytical Method for Phosphatidylcholine

A validated high-pressure liquid chromatography (HPLC) method to determine the contents of PC by HPLC with gradient elution using external standard calibration was used for quantifying surfactant distribution in the set-up compartments [17]. The method is sensitive enough to determine PC in the concentration range from 20 to 2100 µg/mL for *Poractant alfa*, provided that a dual-wavelength detector (WATERS 2487, Waters Corporation, Milford, MA, US) is used. If necessary, samples were diluted to fit within the aforementioned concentration range.

### 2.5. Particle Size Characterization

Particle size distribution was determined by laser diffraction (Helos/BF, Sympatec GmbH, Clausthal-Zellerfeld, Germany). To investigate the consistency over time of the surfactant aerosols generated by the customized eFlow Neos nebulizer, the particle size distribution was determined at different intervals during the continuous nebulization of 13.11 mL, the equivalent *Poractant alfa* volume corresponding to a 600 mg/kg dose for a 1750 g preterm infant. The customized eFlow-Neos was filled with undiluted surfactant and it was continuously nebulized towards the detection area. The aerosol cloud was analysed after nebulization of 200 mg/kg (4.37 mL), after nebulization of 400 mg/kg (8.72 mL) and, finally, after nebulization of 600 mg/kg (13.11 mL). Laser diffraction experiments were conducted at 37 °C, at a relative humidity of 90% ± 5. Each of these experiments was repeated five times using independent nebulizer units. The mass median diameter (MMD), geometric standard deviation (GSD), fine particle fraction and total surfactant output rate (TOR) were determined. The fine particle fraction represents the fraction of particles with a diameter below 5 µm. The TOR represents the total amount of surfactant nebulized in one min (in mg/min).

### 2.6. Statistical Analysis

Comparisons of surfactant deposition between the different set-ups and dosage regimes were analyzed by Student’s t-test with Levene’s test for equality of variances (significance level *P* < 0.01). The correlation between the nebulization time and the lung dose with the nominal dose was assessed by linear regression analysis.

## 3. Results

### 3.1. Nebulizer Positioning Significantly Affects the Lung Dose (LD)

**Figure 2:**
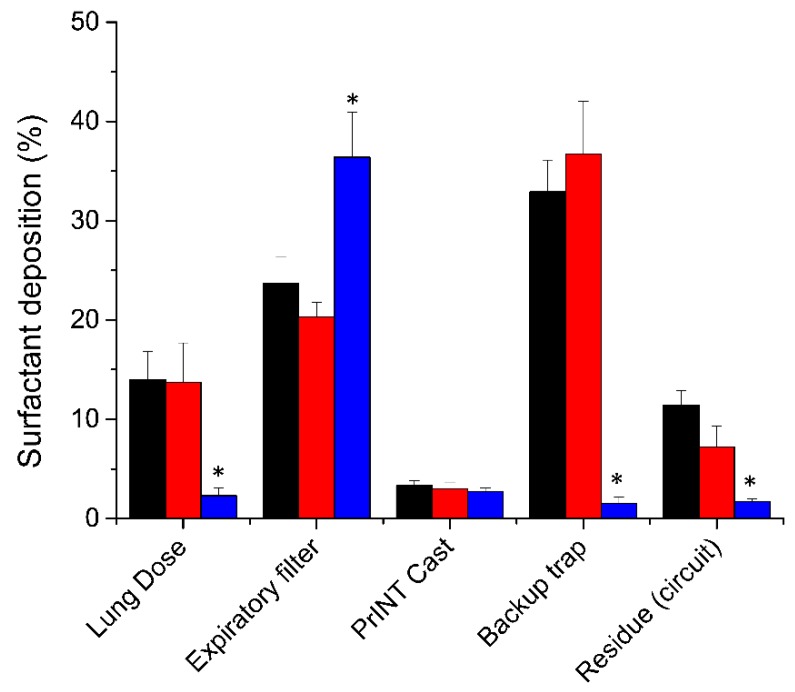
Mean cumulative percentage of deposited surfactant within different set-up compartments placing the nebulizer 7 cm away from the Y-piece (blue bars, *n* = 5) or between the Y piece and the continuous positive airway pressure (CPAP) interface (black bars for nasal mask and red bars for nasal prongs, *n* = 5). * *P* vs. nebulizer placed between the Y-piece and the CPAP interface < 0.01.

The LD registered after placing the nebulizer between the Y-piece and the nasal mask was 14% ± 2.8 of the nominal dose (200 mg/kg) (Figure 2). Remarkably, placing the nebulizer just 7 cm away from the Y-piece reduced the LD by seven-fold to 2.3% ± 0.8 (*P* < 0.01). Placing the nebulizer far from the Y-piece significantly reduced the amount of surfactant detected in the backup trap (1.5% ± 0.7 vs. 32.9% ± 3.2, *P* < 0.01) and in the circuit (1.7% ± 0.3 vs. 11.4% ± 1.5, *P* < 0.01), and significantly increased the amount of surfactant aerosol captured at the expiratory filter (36.4% ± 4.5 vs. 23.7% ± 2.7, *P* < 0.01).

### 3.2. The Choice of CPAP Interface Does not Affect the Surfactant Lung Dose

Irrespective of the CPAP interface used, if the nebulizer was placed between the Y-piece and the CPAP interface, the mean surfactant lung dose was around 14% of the nominal dose (nasal mask 14% ± 2.8, nasal prongs 13.7% ± 4) (Figure 2). The highest fraction of deposited surfactant was detected in the backup trap, which was placed between the PrINT cast and the inspiratory filter. This indicates that a relatively high surfactant aerosol impaction occurs onto the walls of the circuit and CPAP interface right after aerosol generation. The amount of surfactant detected in the backup trap was slightly but not significantly higher with the nasal prongs compared to the nasal mask (36.7% ± 5.3 vs. 32.9% ± 3.2). Conversely, marginal statistical differences were found for the mean surfactant amounts detected in the expiratory filter (23.7% ± 2.7 vs. 20.3% ± 1.5, *P* = 0.047) or deposited within the circuit (11.4% ± 1.5 vs. 7.2% ± 2.1, *P* = 0.011), which were higher when the nasal mask was used.

### 3.3. Lung Dose after Surfactant Nebulization Correlates with the Nominal Dose

**Figure 3:**
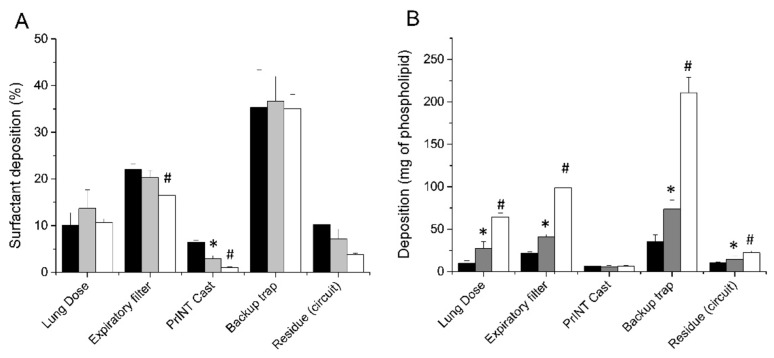
(**A**) Mean cumulative percentage of deposited surfactant within different set-up compartments after delivering surfactant doses of 100 mg/kg (black bars), 200 mg/kg (grey bars), and 600 mg/kg (white bars). (**B**) Mean surfactant mass deposited within the different set-up compartments after delivering surfactant doses of 100 mg/kg (black bars), 200 mg/kg (grey bars), and 600 mg/kg (white bars). * *P* vs. 100 mg/kg dose < 0.01. # *P* vs. 200 mg/kg dose < 0.01.

Three different nominal doses of surfactant, 100, 200 and 600 mg/kg, were delivered in the neonatal CPAP circuit with the customized eFlow Neos nebulizer placed between the Y-piece and the nasal prongs. If the lung dose is expressed as a percentage of the nominal dose, no significant differences are detectable between the different nominal dose regimes (Figure 3A). The mean LDs were 10.1% ± 2.7 (range 9%–13.9%), 13.7% ± 4 (range 10.2%–19.8%), and 10.7% ± 0.8 (range 9.6%–11.5%) for 100, 200 and 600 mg/kg nominal doses, respectively. It is noteworthy that, regardless of the nominal dose, about one-third of the nebulized surfactant was detected within the backup trap. In addition, 20% of the surfactant aerosol was collected in the expiratory filter and less than 10% deposited within the circuit or remained in the nebulizer (residue circuit).

**Figure 4:**
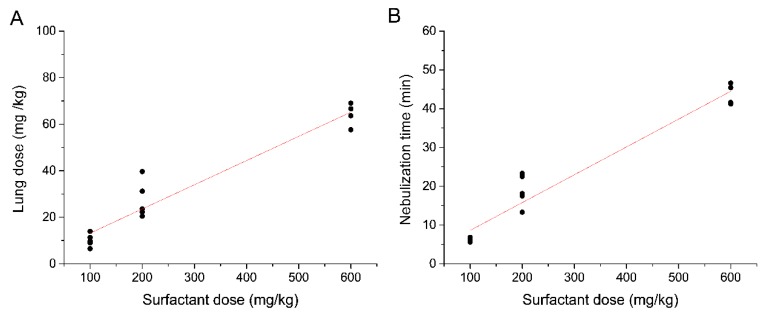
(**A**) Linear regression analysis between the lung dose and the nominal surfactant dose (r^2^ = 0.9256) and (**B**) between the nebulization time and the nominal surfactant dose (r^2^ = 0.9427).

The mean total LDs expressed in mg were 10 ± 2.7, 27.4 ± 7.9, and 64.2 ± 4.92 mg for the 100, 200, and 600 mg/kg nominal doses, respectively (Figure 3B). The LD, expressed as mg/kg, showed a strong linear correlation with the nominal dose (r^2^ = 0.9256) (Figure 4A). The mean nebulization times to deliver nominal surfactant doses of 100, 200, and 600 mg/kg were 6.1 ± 0.5, 18.9 ± 4.1, and 43.7 ± 2.1 min, respectively. Individual nebulization times were also strongly correlated with the nominal dose (r^2^ = 0.9427), indicating a robust performance of the nebulizers over time (Figure 4B).

### 3.4. The Customized eFlow Neos Nebulizer Shows a Consistent Surfactant Aerosol Production Over Time

**Figure 5:**
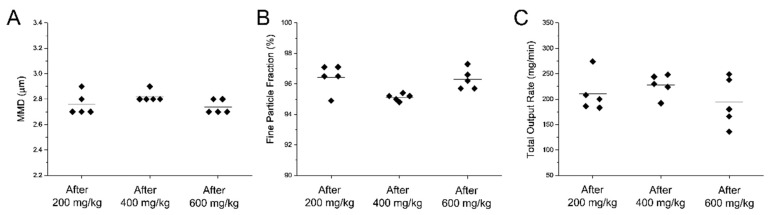
Individual (**A**) mass median diameter (MMD), (**B**) fine particle fraction and (**C**) total output rate values determined at different time-points during the nebulization of a 600 mg/kg dose of *Poractant alfa* (calculated for a birth weight of 1750 g). All parameters were determined after the nebulization of the first 200 mg/kg, after nebulization of 400 mg/kg and after nebulization of 600 mg/kg. The black horizontal line represents the mean of five repetitions.

To investigate the performance of the customized eFlow Neos nebulizer when delivering surfactant aerosols over time, the particle size distribution was characterized by laser diffraction at three different time-points along the nebulization of a total of 1050 mg (13.12 mL) of surfactant, which corresponds to a 600 mg/kg dose for an infant of 1750 g. The mean MMD was 2.76 ± 0.1μm after the nebulization of the first 350 mg of surfactant, 2.8 ± 0.04 μm after nebulizing 700 mg of surfactant and 2.74 ± 0.05 μm after nebulizing 1050 mg of surfactant (Figure 5A). The GSD remained constant at 1.5. The mean fine particle fraction remained constant at each defined time-point of the nebulization process ranging between 95% and 96.5%. (Figure 5B).

Similarly, the TOR was also consistent over time (Figure 5C). The mean TOR was 210 ± 37 mg/min for the nebulization of the first 350 mg of surfactant and remained fairly constant for the remainder of the nebulization process (227 ± 22 and 193 ± 48 for the nebulization of additional 350 mg of surfactant each time).

## 4. Discussion

A non-invasive delivery of nebulized surfactant has been a long-pursued goal in neonatology. A few clinical studies have investigated the safety and efficacy of nebulized surfactant [7,8,9,10,11]. Nevertheless, only two studies could show mild benefits associated with the therapy. Jorch et al. observed an improvement of arterial oxygenation and a reduction of the partial pressure of carbon dioxide after delivering a fixed dose 150 mg of *Bovactant* (natural surfactant) to 28–35 weeks old gestational age infants [7]. More recently, Minocchieri et al. have found a reduction of the need for intubation in the first three days of life in infants with mild RDS (gestational age: 29–35 weeks) who received nebulized *Poractant alfa* [11]. Unfortunately, all these clinical studies are difficult to compare because they used different types of nebulizers (jet and vibrating membrane nebulizers), different surfactant preparations (natural and synthetic) and administration protocols, and rather heterogeneous patient populations. All these studies, however, have highlighted the safety of the therapy and contributed to understanding the complexity of delivering medical aerosols to preterm infants.

The main aim of nebulized surfactant in RDS is to achieve a relatively high lung deposition that will further allow reverting the respiratory distress. Nevertheless, compared to adults, lung deposition in infants is extremely low [19]. Lung deposition in spontaneously-breathing infants has been estimated to be less than 1% of the nominal dose using jet nebulizers, ultrasonic nebulizers or pressurized metered-dose inhalers [20,21]. Fortunately, the use of newer nebulization technologies such as vibrating-membrane nebulizers has been shown to increase lung deposition in neonatal animal models [22,23]. Compared to jet and ultrasonic nebulizers, vibrating-membrane devices generate aerosols without adding airflow to the ventilation circuit and allow delivering large doses at high rates without heating the medication.

In this study, we used an eFlow Neos nebulizer adapted to preterm infants. This technology can nebulize undiluted *Poractant alfa* at relatively high aerosol production rates without altering the surface tension reducing properties of surfactant [17,24,25]. Placing the nebulizer between the Y-piece and the CPAP interface significantly increased the LD compared with placing it at the feeding pipe of the Flexitrunk ventilation system, 7 cm away from the Y-piece. Using an in vitro set-up of pediatric and adult positive pressure ventilation, Ari et al. found out that placing a jet nebulizer in the inspiratory limb, far from the Y-piece, improved the lung dose of salbutamol compared to placing the nebulizer close to the Y-piece [14]. The authors speculated that generating the aerosols within the inspiratory limb produced an aerosol reservoir that was later pushed towards the patient by each mechanical breath. Conversely, we found a significantly reduced LD if surfactant aerosols were generated 7 cm away from the Y-piece. This phenomenon is best explained by the use of CPAP in our circuit instead of using positive pressure ventilation. Generating surfactant aerosols into the inspiratory limb during CPAP most likely diluted the surfactant aerosols within the airflow [12]. We used an airflow of 5 L/min to feed the CPAP circuit, whereas the premature infant respiratory pattern programmed in the breath simulator yielded a minute ventilation (V_M_) of 0.623 L/min. Therefore, just 12% of the air entering the CPAP circuit could ultimately reach the lung dose filter, which obviously had a significant impact on the LD. Accordingly, placing the nebulizer away from the Y-piece showed a reduced LD and was associated with a significantly higher amount of surfactant captured at the expiratory filter. Conversely, placing the nebulizer between the Y-piece and the CPAP interface achieved a 7-fold greater LD. Nevertheless, placing the nebulizer at this position resulted in a high surfactant impaction at the CPAP interface, as denoted by the abundant amount of surfactant collected by the backup trap.

Nasal prongs and nasal masks are widespread used patient-ventilation interfaces in the context of NIV [26]. We did not find significant differences in the surfactant LD after comparing their use in our neonatal ventilation circuit, which indicates that both interfaces are suitable to perform aerosol therapy. Nonetheless, a slightly higher amount of surfactant was detected in the backup trap using the nasal prongs, whereas a marginally significant higher amount of surfactant was detected in the expiratory filter using the nasal mask. Taking together, these findings suggest that the geometry of the nasal mask may offer an extra little space for the aerosol cloud to expand, which slightly reduced the impaction of surfactant droplets within the interface, and promoted a greater surfactant loss during exhalation. Anyhow, irrespective of the CPAP interface used, if the nebulizer was placed between the Y-piece and the CPAP interface, delivering a nominal surfactant dose of 200 mg/kg surfactant, yielded a LD of approximately 28 mg/kg. According to our previous experience with the customized eFlow Neos nebulizer, a nominal dose of 200 mg/kg of surfactant achieved a lung deposition ranging between 8% and 27% in healthy piglets managed with non-invasive ventilation, as estimated from gamma scintigraphy images [27]. The same nominal dose reverted the respiratory distress induced by repeated broncho-alveolar lavages in a rabbit model of acute respiratory distress syndrome [17]. However, in a setting of primary surfactant deficiency (e.g., severe RDS), higher intrapulmonary doses may be eventually required to fully restore lung function [28].

Therefore, we further investigated the surfactant LD after delivering nominal doses ranging from 100 to 600 mg/kg. Across the range of surfactant doses tested in our in vitro set-up, we found a strong linear correlation between both nebulization time and the LD with the nominal dose. These observations indicate a robust performance of the customized eFlow Neos nebulizer with undiluted surfactant. Clinically-available surfactant preparations are viscous suspension composed of phospholipids and peptides [29], which might significantly affect the performance of the nebulizers. In the case of vibrating membrane nebulizers, such phospholipid suspensions can clog the pores of the membrane [30]. For instance, Linner et al. reported the need to dilute *Poractant alfa* 1:1 with saline to achieve the optimal performance of an investigational eFlow vibrating membrane nebulizer [23]. Finer et al. found marked variability in dose output of the single AeroNeb Pro vibrating-membrane nebulizer units when delivering *Lucinactant*, a protein-containing synthetic surfactant, at a concentration of 20 mg/mL [10]. Remarkably, the customized eFlow Neos nebulizer showed a consistent surfactant aerosol production over time. Besides, aerosol characteristics in terms of particle size and fine particle fraction remained unchanged after the nebulization of surfactant doses up to 600 mg/kg.

Further steps have been taken in the preclinical arena with the aim to improve surfactant lung deposition. Previous studies demonstrated that intratracheal nebulization of surfactant to intubated animals using inhalation catheters could achieve an equivalent pulmonary outcome compared with the intratracheal instillation [31,32] but with more gradual hemodynamic changes [33]. Taking these observations as a starting point, different strategies for intracorporeal, yet supraglottic (i.e., non-invasive), atomization have been developed [15,34,35,36]. In this regard, Linner et al. have recently reported a 40% lung surfactant deposition after delivering a 200 mg/kg dose of technetium-labeled *Poractant alfa* to healthy, neonatal piglets managed with CPAP [37]. The key to this remarkable result was most likely the synchronization between the aerosol generator and the inspiratory effort of the animal. Besides, the lung deposition of synthetic surfactant aerosols has also been addressed in vivo. Gregory et al. found a surfactant lung deposition of 11.4% in non-human primates ventilated with CPAP [38]. In this study, *Lucinactant* aerosols were generated with a prototype of a heated capillary aerosol generator. Aerosols were delivered to the ventilation circuit through a device (Afectair^®^) that replaces the Wye connector and is designed to shield the aerosol flow from the CPAP flow. New aerosol technologies have also been applied to the aerosol delivery of synthetic surfactants formulated as dry powders. After the observation that hygroscopic growth could form surfactant aggregates and clog the airways, Pohlmann et al. designed a Continuous Powder Aerosolizer (CPA), which included a humidification step in the aerosol generation process to avoid surfactant aggregation [39]. The device was used to generate recombinant surfactant protein-C (rSP-C) surfactant aerosols, which were safely delivered to preterm lambs but achieved a modest lung deposition and no signs of pulmonary improvement [40]. More recently, Walther et al. have reported a series of spray-dried surfactant formulations to be delivered by a novel aerosolization technology consisting of a cylindrical chamber that can deliver spray-dried surfactant aerosols from a perforated capsule [41]. This device requires flow rates ranging between 4 and 10 L/min for the aerosol generation. This formulation-device combination yielded encouraging results improving the arterial oxygenation in two animal models of respiratory distress either managed with mechanical ventilation or CPAP. These technologies still require further development until they can be assessed for human use.

In summary, this study evidences the convenience of using in vitro set-ups mimicking relevant clinical NIV conditions as a tool to optimize the lung deposition of nebulized surfactant. Placing the nebulizer between the Y-piece and the patient interface improves the surfactant LD compared to placing it at the inspiratory limb of the NIV circuit. Interestingly, relatively high surfactant LDs can be achieved indistinctly using nasal prongs or nasal mask. Most importantly, the investigational vibrating-membrane nebuliser used in this study showed a consistent surfactant aerosol production rate and maintained the aerosol characteristics unchanged—even after having delivered high doses of undiluted *Poractant alfa* at a concentration of 80 mg/mL. However, this investigation has also some limitations that must be acknowledged. In the first place, this is an in vitro study and therefore, the results must be interpreted carefully; for instance, unlike in clinical practice, we tightly sealed the nasal prongs and the mask to the PrINT cast with silicone to keep a close NIV circuit. Secondly, we placed a backup trap between the PrINT cast and the inspiratory filter to collect the aerosol impacting within the interface that moves forward as a liquid. Approximately one-third of the nominal dose was collected by the backup trap, which represents a significant amount of surfactant. Of note, this fraction is expected to follow gravity and could either be swallowed or cause surfactant accumulation within the airways. Therefore, if high surfactant doses are continuously delivered, special care must eventually be taken to avoid surfactant accumulation within the airways [17].

## 5. Conclusions

The management of neonatal RDS is evolving towards the choice of less invasive respiratory support as a primary mode of treatment. In such context, drug nebulization is particularly intriguing. The inclusion of the PrINT model within a realistic in vitro neonatal ventilation circuit is a valuable tool in order to replicate similar conditions to those expected in the clinical practice. Our in vitro set-up allowed us obtaining information on the delivered surfactant lung dose that cannot be easily gained in patients. The present work has demonstrated that device positioning within the respiratory circuit is critical in order to increase the surfactant lung dose.
